# Divergent allometric strategies and biomass allocation patterns of four key tree taxa in subtropical China

**DOI:** 10.3389/fpls.2026.1855123

**Published:** 2026-06-29

**Authors:** Jia Yang, Wencheng Peng

**Affiliations:** Hainan Academy of Forestry (Hainan Academy of Mangroves), Haikou, China

**Keywords:** allometric scaling, bark defense, biomass additivity, life-history strategy, resource allocation, subtropical forests

## Abstract

**Introduction:**

Accurate forest biomass estimation must simultaneously satisfy the statistical additivity of components and resolve the resource-allocation trade-offs that unfold during tree ontogeny, yet conventional models often fail on both counts and overlook bark as a distinct functional component.

**Methods:**

Using destructive-sampling data from 498 trees of *Cunninghamia lanceolata*, *Pinus massoniana*, *Populus deltoides*, and an oak-dominated Fagaceae complex (*Quercus* spp.) in subtropical China, we developed a compatible biomass equation system that resolves stem wood, bark, branches, and leaves separately. Component additivity was enforced through Nonlinear Seemingly Unrelated Regression (NSUR), which exploits the cross-equation error covariance, using component-specific predictors (DBH or the compound term D²H), and critical allometric thresholds were identified by derivative analysis of the stem mass fraction.

**Results:**

Incorporating D²H improved the stem-wood fit, and the additive system explained 93.6–98.4% of the variance in total aboveground biomass. Allometric trajectories revealed divergent strategies. *P. deltoides* and the conifers followed a “vertical-maximization” strategy, marked by high stem-wood scaling exponents (b ≈ 0.94–0.99) and early stem dominance; within this group *P. deltoides* showed a broad structural-adjustment phase (DBH 3.9–13.9 cm), whereas *C. lanceolata* was developmentally rigid. In contrast, *Quercus* spp. followed a “structural-persistence” strategy, maintaining high branch allocation and a rare near-isometric bark scaling (b ≈ 1.0), which contrasts with the diminishing marginal investment in conifer bark (b ≈ 0.80–0.87, i.e. b< 0.9).

**Discussion:**

Because hardwood bark scales near-isometrically rather than as a constant fraction, ignoring its allometry systematically underestimates carbon stocks in mature broadleaved forests. The resulting taxon- and component-specific equation system provides a directly applicable tool for reducing systematic bias in regional carbon assessment, indicating that carbon accounting should move beyond a purely volumetric perspective toward a component-resolved framework that incorporates life-history allocation strategies.

## 1 Introduction

In the context of global climate governance, the precise quantification of forest carbon sinks has shifted from simple estimates of growing stock to a deeper analysis of ecosystem functional processes (Mo et al., 2023; Liang et al., 2022; Friedlingstein et al., 2025; Migliavacca et al., 2025). Although generalized allometric equations provide a convenient tool for large-scale carbon assessment, they often introduce unquantifiable systematic biases by neglecting the interplay between species-specificity and site conditions (Chave et al., 2014; Kaushal et al., 2022). This issue is particularly acute in highly heterogeneous subtropical regions, where remote sensing inversion models and Dynamic Global Vegetation Models (DGVMs) urgently require physical calibration against high-quality destructive sampling data (Pan et al., 2011; Forkel et al., 2019). Therefore, developing localized, species-specific, high-precision biomass models remains a physical cornerstone for reducing uncertainties in regional carbon budgets.

However, current biomass quantification systems are constrained by two major methodological bottlenecks: a lack of statistical additivity and insufficient component resolution. Conventional modeling often fits equations for each organ (stem, branch, leaf) independently, leading to a “sum-of-parts” paradox where component sums do not equal the total, severely undermining model credibility (Parresol, 2001; Zhang and Borders, 2004; Wang et al., 2023). While Nonlinear Seemingly Unrelated Regression (NSUR) has provided a mathematical breakthrough in ensuring additivity, its application is frequently limited to coarse, binary divisions of “stem/crown,” widely neglecting the independent quantification of bark—a key functional trait (Paine et al., 2010; Pati et al., 2022). Bark not only serves as a tree’s primary defense against fire and pathogens (Franceschi et al., 2005) but also exhibits divergent allometric trajectories among functional types. Because bark can constitute a substantial yet taxon-variable fraction of stem mass, treating it as a constant proportion of wood propagates a systematic, functional-type-dependent error into stem and total biomass estimates. Neglecting the allometric specificity of bark can lead to a significant underestimation of carbon stocks in mature forests (Rosell, 2016). Thus, simultaneously resolving statistical additivity and the quantification of fine-scale components, especially bark, is crucial for transitioning from coarse estimation to precision management of carbon sinks.

More fundamentally, allometric relationships are not merely mathematical expressions of biomass but are outcomes of evolutionarily shaped resource-allocation strategies (Enquist and Niklas, 2002; Zhou et al., 2021). While metabolic scaling theory predicts that plants should adhere to fixed exponents to optimize energy use, empirical evidence reveals that trees dynamically adjust carbon investment among mechanical support, hydraulic transport, and photosynthetic acquisition in response to light competition and hydraulic constraints during their development (Poorter et al., 2012; Badel et al., 2015). This ontogenetic drift manifests in starkly different trajectories: fast-growing conifers may adopt an aggressive “vertical-maximization” strategy, whereas slow-growing hardwoods like oaks may pursue a conservative “structural-defense” strategy (Guo et al., 2025). Regrettably, existing research has predominantly focused on fitting static equations (Balbinot et al., 2018; Zhu et al., 2025), lacking a dynamic analysis of the resource allocation trade-offs throughout the life-history of key subtropical tree species. This gap limits our mechanistic understanding of forest ecosystem adaptability.

To overcome these limitations, this study leverages extensive destructive sampling data from 498 trees of *C. lanceolata*, *P. massoniana*, *P. deltoides*, and *Quercus* spp. from subtropical China to address two core issues in mathematical modeling and ecological mechanisms. We aimed to: (1) develop a statistically compatible biomass model system that includes fine-scale components like bark by incorporating tree height as a compound predictor and applying NSUR, thereby eliminating the logical paradoxes and blind spots of traditional methods; and (2) quantify the dynamic “growth-defense” trade-offs during ontogeny by defining allometric thresholds through derivative analysis. This research establishes a mathematical benchmark for precise regional carbon accounting and provides a theoretical basis for multi-objective forest management from a life-history strategy perspective.

## 2 Materials and methods

### 2.1 Study area

This study was conducted in Hunan Province, China (108°47′–114°15′ E, 24°38′–30°08′ N), a region central to the country’s mid-subtropical zone, within the humid subtropical belt of China (approximately 24–30° N). Its distinctive horseshoe-shaped geomorphology, defined by mountains to the east, south, and west, creates significant habitat heterogeneity and vertical climatic gradients, making it a suitable setting for assessing allometric plasticity across site conditions (Shen et al., 2022).

The region experiences a humid subtropical monsoon climate, with a mean annual temperature of 16.0–18.0 °C and annual precipitation of 1200–1800 mm (Wang et al., 2024). This climate’s pronounced hydrothermal synchrony during the growing season drives high net primary productivity. The zonal soils are primarily Ultisols and Oxisols, developed from parent materials such as granite, slate, and Quaternary red clay. As a pivotal region for forestry in southern China, Hunan has long maintained some of the nation’s highest stocking volumes and coverage of *C. lanceolata* and *P. massoniana*. The prevalence of these species, alongside fast-growing *P. deltoides* plantations and secondary *Quercus* spp. forests, ensures that our study area encompasses a full functional spectrum of taxa. Biomass models developed from this region are therefore highly representative of growth patterns throughout China’s subtropical plantation ecosystems.

### 2.2 Data collection and processing

**Figure 1:**
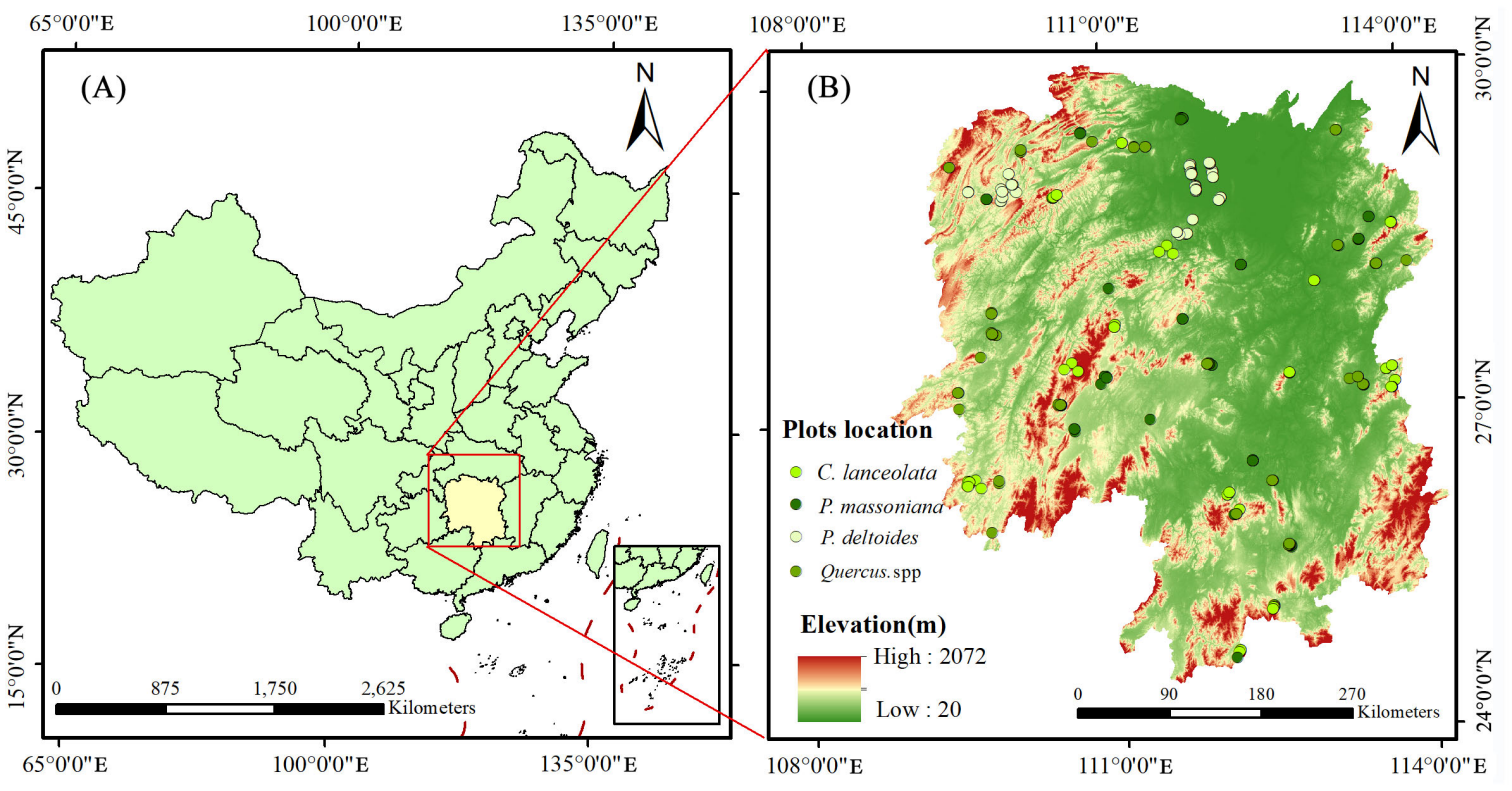
Geographic distribution of the destructive-sampling sites across Hunan Province, China. Markers denote sampling locations within the 14 sampled prefectures, color-coded by tree taxon (*C. lanceolata*, *P. massoniana*, *P. deltoides*, and *Quercus* spp.). The inset locates Hunan Province within the humid subtropical zone of China.

We employed a stratified sampling strategy to cover 14 prefectures across Hunan Province (**Figure 1**), spanning a habitat gradient from the northwestern mountains to the northern plains to capture regional environmental heterogeneity. The sampled stands included a complete chronosequence from young to over-mature forests, balanced across different site indices and management intensities. A total of 498 trees were destructively sampled, comprising *C. lanceolata*, *P. massoniana*, *P. deltoides*, and *Quercus* spp. represented primarily by *Q. glauca*. No individuals were excluded after field sampling; all destructively sampled trees with a complete set of component dry-mass measurements were retained for model development. The samples were evenly distributed across a wide range of diameter at breast height (DBH; 3.0–35.3 cm) and tree height (2.8–29.1 m), ensuring they were representative of the population growth characteristics of major plantation species in the region (**Table 1**).

**Table 1:** Descriptive statistics of dendrometric characteristics for the four taxa. N, sample size; DBH, diameter at breast height; SD, standard deviation.

Taxon	N	DBH (cm)		Height (m)		Total biomass (kg)	
		Mean ± SD	Range	Mean ± SD	Range	Mean ± SD	Range
*C. lanceolata*	130	20.4 ± 7.2	3.0–35.3	14.5 ± 5.2	2.8–25.6	122.7± 99.4	0.9–417.0
*P. massoniana*	118	16.9 ± 8.4	3.5–32.6	13.4 ± 5.0	3.9– 25.3	126.1 ± 119.1	1.5–455.4
*P. deltoides*	123	16.4 ± 8.3	3.6– 32.0	16.2 ± 6.6	5.3– 29.1	101.8 ± 100.8	1.2–355.5
*Quercus* spp.	127	15.8 ± 8.6	3.5– 32.5	12.4 ± 4.3	3.7– 21.8	159.6 ± 165.7	3.1–621.1

Field measurements followed the full-harvest standard for biomass determination. We dissected the aboveground portion of each tree into four independent components: stem wood, bark, branches, and leaves. For the stem, we used the sectional method, cutting discs at 2 m intervals from the base. Wood and bark were separated and weighed fresh in the field to quantify the fresh mass allocation at different heights. For the crown, we collected standard branch and leaf samples from the upper, middle, and lower canopy layers. All samples were transported to the laboratory and oven-dried at 85 °C to a constant weight. The dry mass of each component was calculated based on the total fresh mass and the dry-to-fresh mass ratio. Total aboveground biomass (AGB) was defined as the algebraic sum of the four component dry masses. This procedure ensured physical additivity in the raw data, establishing a benchmark for subsequent assessments of statistical compatibility among different modeling algorithms.

### 2.3 Compatible biomass model construction

#### 2.3.1 Base model variable selection

Based on the principles of biological allometry (Tahvanainen and Forss, 2008; Sileshi et al., 2023), we first selected the optimal predictor variables for each tree component independently. To investigate the gain in explanatory power from incorporating tree height (H). we compared three typical non-linear base models: a power function with DBH (D) as the sole predictor (Model I: $W=\beta_{0}D^{\beta_{1}}$), a compound variable model reflecting stem geometry (Model II: $W=\beta_{0}{\left(D^{2}H\right)}^{\beta_{1}}$), and a two-variable model accounting for the independent effects of DBH and height (Model III: $W=\beta_{0}D^{\beta_{1}}H^{\beta_{2}}$). Based on the statistical performance evaluated by the Akaike Information Criterion (AIC) and Root Mean Square Error (RMSE), combined with the biological plausibility of the parameter estimates, we determined the optimal base equation structure for each biomass component of each tree taxon. Full goodness-of-fit statistics — residual sum of squares (RSS), mean absolute error (MAE) and AIC — for all three candidate models across every taxon–component combination are provided in **Supplementary Table 1**, enabling readers to judge whether the selected form represents a substantive improvement over the simpler alternatives.

#### 2.3.2 Simultaneous equations and statistical compatibility

To address the logical paradox of “the sum of the parts not equaling the whole” that arises from fitting component equations independently, we constructed a system of non-linear simultaneous equations based on component additivity. Within this framework, the total aboveground biomass equation is not fitted independently; instead, it is strictly defined as the algebraic sum of the four component predictions, thereby enforcing statistical compatibility in the model structure. The system is specified in Equation 1:

(1) $$\{\begin{array}{c} W_{i}=\beta_{i0}X_{i}^{\beta_{i1}}+\epsilon_{i}\left(i=\text{wood, bark, branch, leaf}\right)\\ {\hat{W}}_{total}={\hat{W}}_{wood}+{\hat{W}}_{bark}+{\hat{W}}_{branch}+{\hat{W}}_{leaf} \end{array}$$

where *W_i_* is the observed biomass of the *i*-th component, *X_i_* is its optimal predictor, and *ϵ_i_* (*i* = wood, bark, branch, leaf) is the error term of the corresponding component equation. The total-biomass equation carries no independent error term, as it is defined as the exact algebraic sum of the four component predictions. Given that the growth of different organs on the same tree is driven by common genetic and environmental factors, their error terms are often biologically correlated. We therefore employed Nonlinear Seemingly Unrelated Regression (NSUR) for the simultaneous parameter estimation. NSUR was preferred over independent equation-by-equation fitting and over linear seemingly-unrelated regression because the biomass relationships are intrinsically non-linear and additivity must hold across these non-linear equations: NSUR estimates all component equations jointly under the additivity constraint, and its generalized-least-squares step weights the equations by the contemporaneous (cross-equation) error covariance, yielding parameter estimates that are simultaneously strictly additive and asymptotically more efficient than equation-by-equation estimation (Parresol, 2001). This method captures and utilizes the cross-equation error covariance, which improves the asymptotic efficiency and statistical compatibility of the parameter estimates (Rose and Lynch, 2001; Wang et al., 2023). To rigorously evaluate the predictive performance and generalization ability of the model system, we implemented a 10-fold cross-validation procedure. The entire dataset was randomly partitioned into ten mutually exclusive subsets; in each iteration, nine subsets were used for model training, while the remaining subset served as the validation set. We calculated the Mean Error (ME) and Mean Absolute Error (MAE) to quantify the model’s stability and accuracy on independent data.

#### 2.3.3 Heteroscedasticity analysis and weighted correction

Biomass data typically exhibit heteroscedasticity, where the dispersion of residuals increases exponentially with the size of the predictor variable (**Supplementary Figure 1A**). Without correction, parameter estimates from Ordinary Least Squares (OLS) would be disproportionately influenced by large-diameter samples, reducing the model’s predictive sensitivity for smaller individuals. To address this, we introduced an adaptive weight function (*w*) into the parameter estimation, defined as the inverse power of the predictor variable (Equation 2):

(2) $$w_{i}=1/X_{i}^{k}$$

The weighting exponent, *k*, was determined for each component (pooled across all four taxa, i.e. one exponent per component rather than one per taxon–component combination) by fitting a log-linear regression of the squared OLS residuals ( $e_{i}^{2}$) against the predictor variable (*X_i_*). The optimal weighting exponents were calculated as 1.70, 2.34, 3.17, and 5.08 for stem wood, bark, branches, and leaves, respectively. After applying weighted non-linear least squares, the distribution of standardized residuals exhibited a uniform, horizontal trend (**Supplementary Figure 1B**), confirming the elimination of heteroscedasticity and satisfying the assumption of homoscedasticity for regression analysis.

### 2.4 Allometric analysis of biomass allocation

#### 2.4.1 Interspecific differences in allometric scaling

To quantify statistical differences in resource allocation among tree taxa, we performed an Analysis of Covariance (ANCOVA) on the log-transformed, linearized data. Tree taxon was set as the grouping factor and the dendrometric variable as the covariate. We tested the significance of the “taxon × size” interaction term (*P* < 0.05) to determine whether the allometric scaling exponents (slope *b*) differed among taxa. *Post-hoc* multiple comparisons were then used to identify distinct allometric groups.

#### 2.4.2 Allocation patterns and trade-off strategies

We calculated the mass fraction of each component relative to the total aboveground biomass. To analyze the dynamics of resource allocation during development (ontogenetic drift), we fitted non-linear trajectories of these fractions against DBH using Locally Estimated Scatterplot Smoothing (LOESS). To elucidate evolutionary trade-off strategies between organs, we applied Standardized Major Axis (SMA) regression to analyze the bivariate scaling relationships on log-log axes between metabolic (leaf) and support (stem, branches) organs, as well as between defensive (bark) and growth (wood) tissues. The SMA method accounts for natural variation and measurement error in both variables, making it more suitable than ordinary least squares for analyzing such functional trait correlations.

#### 2.4.3 Determination of critical developmental thresholds

Based on the non-linear trajectory of the Stem Mass Fraction (SMF) with increasing DBH, we defined two critical thresholds marking shifts in growth strategy:

**Crossover Point (*D_CP_*):** Based on the theoretical framework of resource allocation trade-offs (Enquist and Niklas, 2002), we operationally defined *D_CP_* as the critical diameter where stem biomass first exceeds the total crown (branch + leaf) biomass. This threshold signifies a fundamental shift in carbon allocation priority from canopy photosynthetic acquisition to stem mechanical support and volume accumulation.

**Stabilization Threshold (*D_ST_*):** Drawing on the asymptotic analysis of forest growth equations (Pretzsch, 2009), we defined *D_ST_* as the diameter where the first derivative (marginal growth rate) of the fitted SMF curve first drops below 1%. The 1% criterion is an operational proxy for the near-asymptotic region of the SMF trajectory: it identifies the diameter at which the marginal change in stem allocation per unit DBH becomes negligible, analogous to the saturation segment that follows the inflection point of sigmoidal forest growth functions (Pretzsch, 2009). This metric objectively quantifies the entry into a “plateau phase,” indicating that the tree has completed its early architectural adjustments and entered a structurally stable phase.

All statistical analyses were performed in the R environment (version 4.2.0).

## 3 Results and analysis

### 3.1 Variable response and parameter characteristics of allometric models

Multi-model selection based on AIC and RMSE revealed distinct biophysical constraints on biomass accumulation at the organ level (**Table 2**). For stem wood, although the three-parameter model (Model III, based on *D*, *H*) exhibited a slightly lower AIC for species like *C. lanceolata*, we prioritized the two-parameter compound variable model (Model II, based on *D^2^H*) as the foundational equation. This decision was based on the principle of parsimony and geometric principles: *D^2^H* serves as a proxy for stem volume. Using Model II ensured a high goodness-of-fit (*R^2^*>0.93) while reducing the number of parameters and avoiding the risk of overfitting due to multicollinearity between DBH and height. This underscores that stem wood biomass is primarily governed by a “volume–density” mechanism.

**Table 2:** Goodness-of-fit comparison of the three candidate base models across tree taxa and components.

Component	Taxon	Model I (*D*)	Model II (*D*^2^*H*)	Model III (*D*, *H*)	Lowest-AIC model
Stem Wood	*C. lanceolata*	1143.2	1122.9	1114.3	Model III
	*P. massoniana*	1097.9	1030.4	1031.3	Model II
	*P. deltoides*	1069.7	928.2	921.7	Model III
	*Quercus* spp.	1223.5	1078.1	1077.5	Model III
Stem Bark	*C. lanceolata*	724.9	743.3	725.2	Model I
	*P. massoniana*	563.7	605.3	563.8	Model I
	*P. deltoides*	547.1	473.0	472.8	Model III
	*Quercus* spp.	907.0	846.9	838.6	Model III
Branch	*C. lanceolata*	897.0	922.0	892.3	Model III
	*P. massoniana*	846.7	898.1	798.5	Model III
	*P. deltoides*	855.0	871.5	852.2	Model III
	*Quercus* spp.	1050.2	1082.8	1050.5	Model I
Leaf	*C. lanceolata*	797.8	812.2	798.0	Model I
	*P. massoniana*	627.8	643.0	586.6	Model III
	*P. deltoides*	517.2	525.6	483.4	Model III
	*Quercus* spp.	744.5	772.7	727.2	Model III

Model forms: Model I, $W=\beta_{0}D^{\beta_{1}}$; Model II, $W=\beta_{0}{\left(D^{2}H\right)}^{\beta_{1}}$; Model III, $W=\beta_{0}D^{\beta_{1}}H^{\beta_{2}}$. Values are Akaike information criterion (AIC) scores (dimensionless; lower is better). Full RSS, MAE, and AIC statistics are given in **Supplementary Table 1**.

The variable response of stem bark, however, showed a clear phylogenetic divergence. Broadleaved species (*P. deltoides*, *Quercus* spp.) exhibited “volume-dependent” growth, for which *D^2^H* was the optimal predictor, implying that bark construction is synchronized with stem volume accumulation. In contrast, the bark biomass of coniferous species (*C. lanceolata*, *P. massoniana*) was better described by the single-variable DBH model (Model I). This indicates that their bark increment is mainly driven by radial expansion and is weakly coupled with height growth. For crown components (branches and leaves), the single-variable DBH model (Model I) was consistently the most parsimonious and effective choice, supporting the pipe model theory’s assumption that the biomass of terminal organs is constrained by sapwood transport area (∝*D*^2^).

The parameters of the compatible equation system, developed on the basis of this component-specific variable-selection scheme, further quantified key life-history trade-offs (**Table 3**). The stem wood scaling exponents (*b*) for angiosperms (*b* ≈ 0.96–0.99) were significantly higher than those for gymnosperms (*b* ≈ 0.94–0.95), indicating that broadleaved species maintain higher wood density or biomass accumulation efficiency for an equivalent increase in volume. Notably, the pioneer species P. deltoides exhibited the highest stem exponent (*b* = 0.9866), reflecting an aggressive carbon investment strategy to rapidly occupy vertical space. In terms of bark allocation, broadleaved species (*b* ≈ 0.91–0.99 based on *D*^2^*H*) displayed nearly isometric and continuous investment. In contrast, coniferous species (*b* ≈ 2.0–2.4 based on *D*, corresponding to strong negative allometry in *D*^2^*H* space) showed a pronounced trend of diminishing marginal investment.

**Table 3:** Parameter estimates and goodness-of-fit statistics for the compatible equation systems of the four taxa.

Component	Predictor (X)	Taxon	Model Coefficients (SE)		Goodness-of-fit		Test effect	
			a	b	R²	RMSE (kg)	ME (kg)	MAE (kg)
Stem Wood	D²H	*C. lanceolata*	0.0187 (0.005)	0.9412 (0.027)^b^	0.938	17.76	-1.775	12.265
	D²H	*P. massoniana*	0.0240 (0.007)	0.9517 (0.030)^b^	0.953	20.02	1.38	12.846
	D²H	*P. deltoides*	0.0119 (0.002)	0.9866 (0.020)^a^	0.982	10.28	-1.927	6.406
	D²H	*Quercus* spp.	0.0289 (0.006)	0.9611 (0.021)^ab^	0.976	16.47	-0.667	11.062
Stem Bark	D	*C. lanceolata*	0.0090 (0.003)	2.3828 (0.099)^a^	0.875	3.84	0.001	2.655
	D	*P. massoniana*	0.0209 (0.007)	2.0644 (0.101)^a^	0.893	2.68	0.001	1.634
	D²H	*P. deltoides*	0.0034 (0.001)	0.9142 (0.021)^b^	0.977	1.61	-0.297	1.053
	D²H	*Quercus* spp.	0.0039 (0.002)	0.9904 (0.050)^b^	0.891	6.63	0.081	3.965
Branch	D	*C. lanceolata*	0.0043 (0.003)	2.6065 (0.197)^a^	0.680	7.45	0.029	5.256
	D	*P. massoniana*	0.0665 (0.030)	1.9759 (0.139)^a^	0.810	9.07	0.080	6.225
	D	*P. deltoides*	0.0402 (0.023)	2.0260 (0.177)^a^	0.732	7.63	0.006	4.697
	D	*Quercus* spp.	0.0704 (0.029)	2.1308 (0.125)^a^	0.839	14.76	-1.11	9.802
Leaf	D	*C. lanceolata*	0.0027 (0.002)	2.5947 (0.228)^a^	0.605	5.09	0.010	3.629
	D	*P. massoniana*	0.0684 (0.053)	1.4364 (0.242)^a^	0.411	3.53	0.029	2.001
	D	*P. deltoides*	0.1270 (0.079)	1.0449 (0.200)^b^	0.297	1.93	0.021	1.312
	D	*Quercus* spp.	0.0350 (0.019)	1.8604 (0.164)^a^	0.677	4.43	-0.014	3.015
Total AGB	Sum	*C. lanceolata*	—	—	0.936	24.97	-1.735	17.681
	Sum	*P. massoniana*	—	—	0.966	22.12	1.489	14.823
	Sum	*P. deltoides*	—	—	0.984	12.53	-2.196	8.800
	Sum	*Quercus* spp.	—	—	0.977	25.16	-1.71	16.927

Model form: *W* = *a*·*X^b^* (*X* = *D* or *D*²*H*); standard errors in parentheses. Different superscript letters within a component column denote significant differences in *b* among taxa (*P* < 0.05). Total AGB is the sum of component predictions; the “Sum” rows report its fit.

Although the leaf biomass model for *P. deltoides* showed a relatively lower fit (*R*^2^ = 0.297), likely due to high crown plasticity, the simultaneous equation system constructed with NSUR effectively captured the cross-component error covariance. Consequently, the predictive accuracy (*R*^2^) for total aboveground biomass remained consistently high, ranging from 0.936 to 0.984. The 10-fold cross-validation results confirmed the system’s robustness: the Mean Error (ME) for total biomass was negligible (e.g., -1.735 kg for *C. lanceolata*), and the Mean Absolute Error (MAE) was well-controlled (e.g., 8.8 kg for *P. deltoides*), demonstrating that the compatible system effectively eliminates cumulative errors and ensures logical consistency in large-scale predictions.

### 3.2 Ontogenetic drift in resource allocation

**Figure 2:**
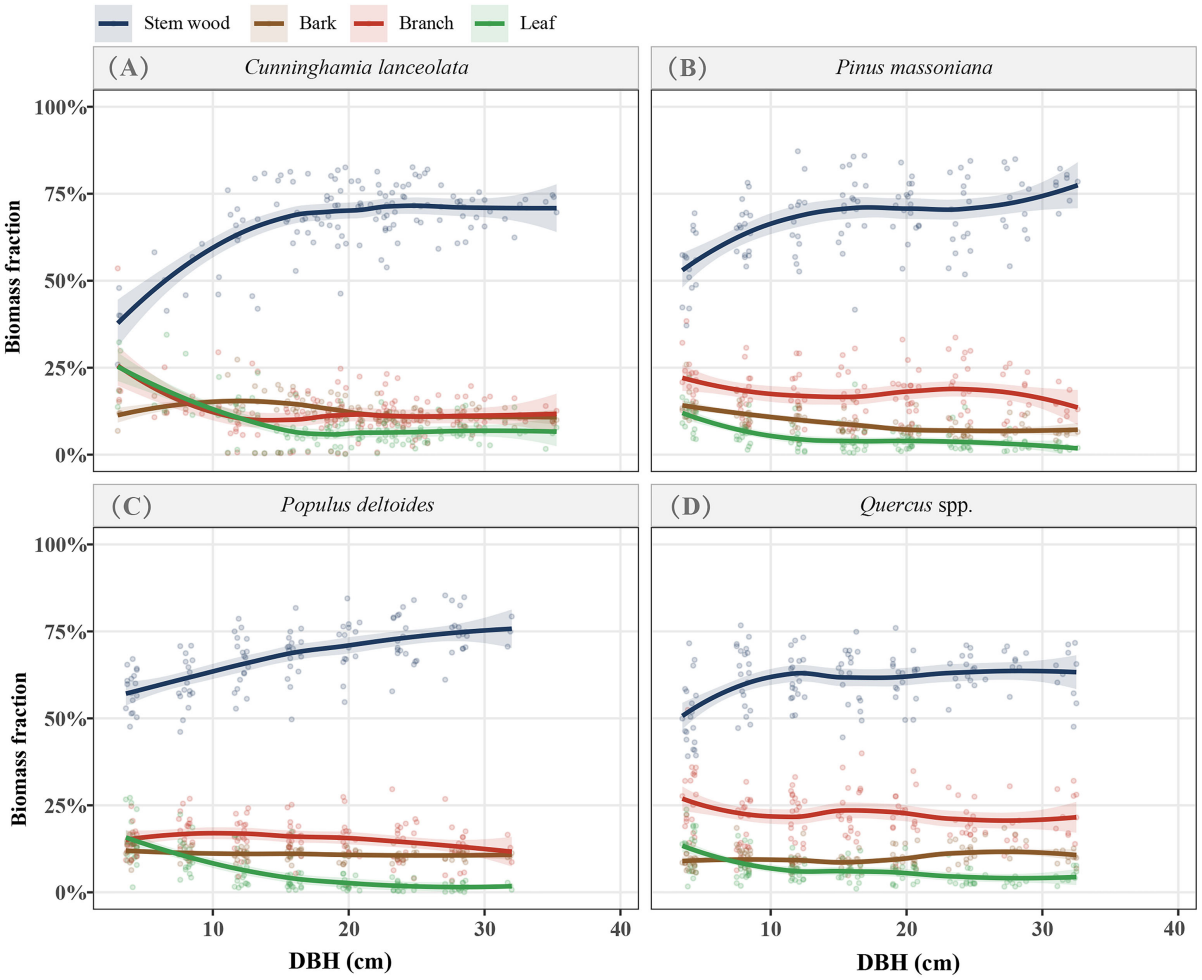
Ontogenetic trajectories of organ biomass allocation for the four taxa: **(A)** Cunninghamia lanceolata, **(B)** Pinus massoniana, **(C)** Populus deltoides, and **(D)** Quercus spp. Points are observed biomass fractions of individual trees; solid lines are LOESS fits with 95% confidence bands. Colors denote components: stem wood (dark blue), bark (brown), branch (red), and leaf (green).

Biomass allocation exhibited a size-dependent ontogenetic drift with increasing DBH, following the universal pattern of shifting resources from photosynthetic organs to structural support organs (**Figure 2**). However, fundamental divergences in the rate of allocation and structural investment were observed among functional types. Coniferous species displayed dramatic architectural remodeling. In *C. lanceolata*, the stem wood fraction surged from below 40% to over 70% within the narrow juvenile phase of DBH 4–15 cm, accompanied by an exponential decay in the leaf biomass fraction. This early establishment of stem dominance reflects a selection priority for vertical space occupancy, minimizing non-productive costs through rapid canopy closure and subsequent self-pruning. *P. deltoides* exhibited a precocious mode of volume accumulation, with its initial stem fraction already exceeding 55% and steadily rising to over 75% with increasing size. This trajectory validates a rapid lignification strategy that maintains high height-to-diameter ratios by suppressing lateral branching and channeling photosynthates to the main stem. In contrast, *Quercus* spp. revealed a unique structural persistence strategy. Its branch biomass fraction remained high and stable at around 20–25% across the entire observed size range—significantly higher than in other taxa. Furthermore, its bark fraction showed a stable, rather than the typical declining, trend in later growth stages. Together, these features confirm that this hardwood trades off part of its stem-accumulation rate for enhanced mechanical safety margins and greater resilience to environmental stress.

### 3.3 Allometric trade-offs between metabolic and support organs

**Figure 3:**
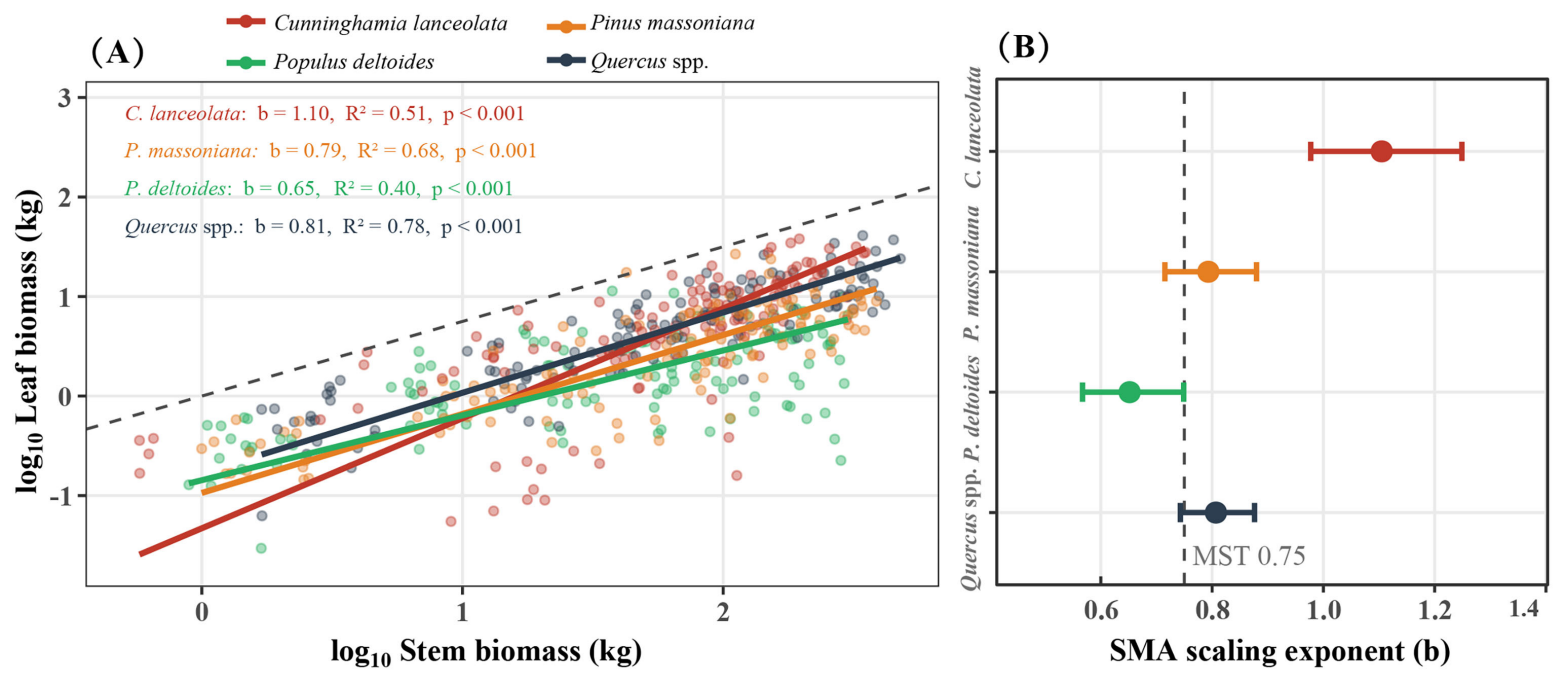
Allometric scaling between metabolic (leaf) and support (stem) biomass for the four taxa. **(A)** Bivariate plots on log_10_–log_10_ axes; solid lines are standardized major axis (SMA), and the dashed line is the 3/4 slope predicted by Metabolic Scaling Theory (MST). **(B)** SMA exponents (b) with 95% confidence intervals; the dashed line marks the MST threshold (b = 0.75).

The log-linear relationship between leaf and stem biomass confirmed the biophysical constraints imposed by pipe-model theory on subtropical tree species (**Figure 3A**), while the divergence in SMA scaling exponents (slope *b*) quantified the trade-off efficiency between photosynthetic gain and structural cost (**Figure 3B**). *P. deltoides* exhibited the lowest exponent (*b* ≈ 0.65), the only taxon whose 95% confidence interval fell entirely below the 3/4 threshold predicted by Metabolic Scaling Theory (MST). This reflects a “diminishing support efficiency” strategy, in which this fast-growing pioneer prioritizes vertical height over crown expansion once the canopy closes. *P. massoniana* (*b* ≈ 0.79) and *Quercus* spp. (*b* ≈ 0.81) lay closest to the MST 3/4 line, with confidence intervals bracketing it, consistent with a dynamic balance between mechanical safety and hydraulic transport that supports a large crown at a near-optimal ratio. By contrast, *C. lanceolata* showed the steepest scaling (*b* ≈ 1.10); however, the comparatively weak leaf–stem correlation in this species (R² = 0.51) widens its confidence interval and inflates the SMA slope, so this departure from 3/4 should be interpreted with caution rather than as evidence of strictly super-proportional crown investment.

### 3.4 Allometric differentiation in bark defense investment

**Figure 4:**
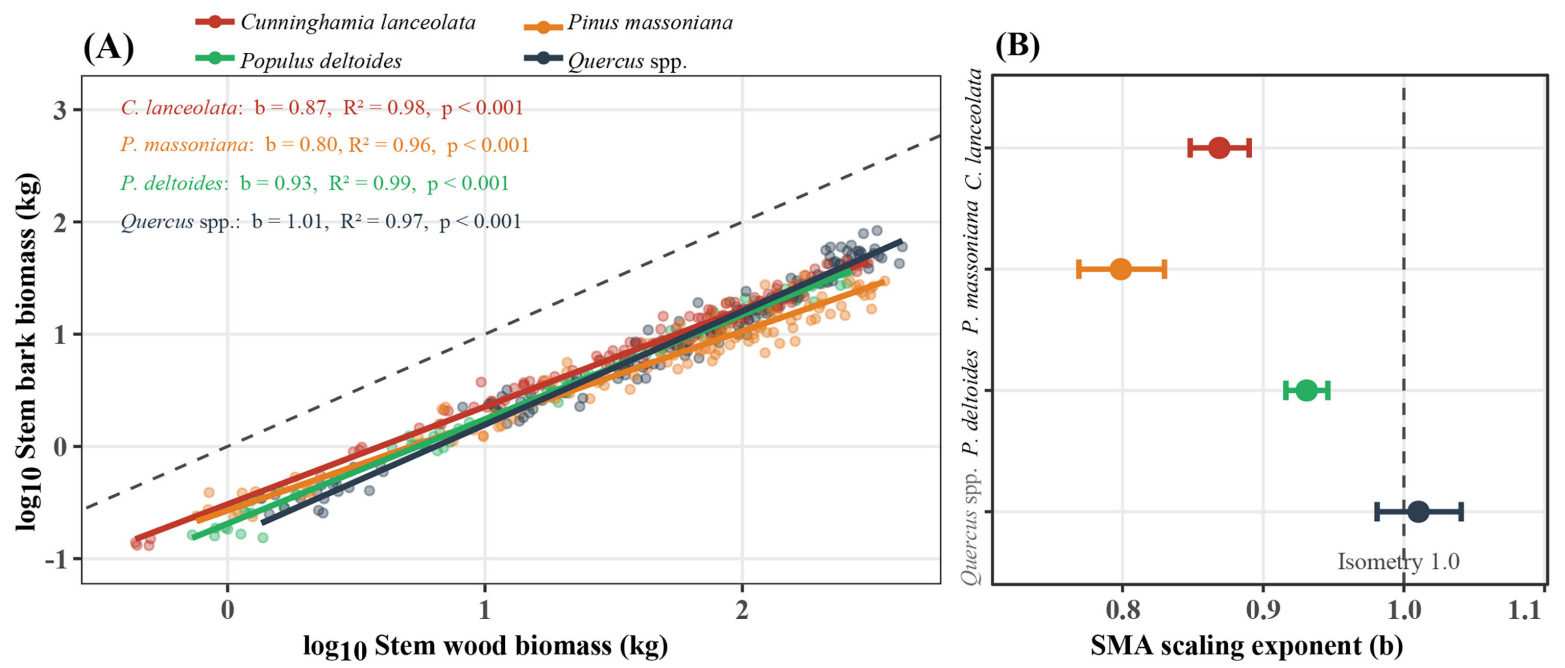
Allometric scaling between defense (stem bark) and growth (stem wood) biomass for the four taxa. **(A)** Bivariate plots on log_10_–log_10_ axes; solid lines are standardized major axis (SMA) fits and the dashed line marks isometry (*b* = 1.0). **(B)** SMA exponents (*b*) with 95% confidence intervals; the dashed line marks the isometric threshold (*b* = 1.0).

The allometric trajectory of bark versus wood biomass quantified the resource trade-off between defense construction and radial growth (**Figure 4A**), while the distinct divergence in scaling exponents (*b*) revealed two contrasting evolutionary strategies (**Figure 4B**). *Quercus* spp. exhibited a rare isometric scaling pattern (*b* ≈ 1.0), indicating that bark accretion remains synchronized with wood accumulation throughout ontogeny. This “structural persistence” strategy involves continuous, high-level carbon investment to thicken the bark, thereby constructing a robust physical barrier against fire or biotic stress. Conversely, coniferous species (*C. lanceolata*, b ≈ 0.87; *P. massoniana*, b ≈ 0.80) displayed significant negative allometry (b< 0.9). This trend of diminishing marginal investment with increasing size reflects a “resource optimization” logic typical of pioneer species: maintaining a high bark ratio in the seedling stage to establish basal defense, but aggressively reducing defense costs in maturity to prioritize carbon allocation towards vertical growth and volume accumulation for light competition in closed canopies.

### 3.5 Division of stand management stages based on allometric thresholds

**Figure 5:**
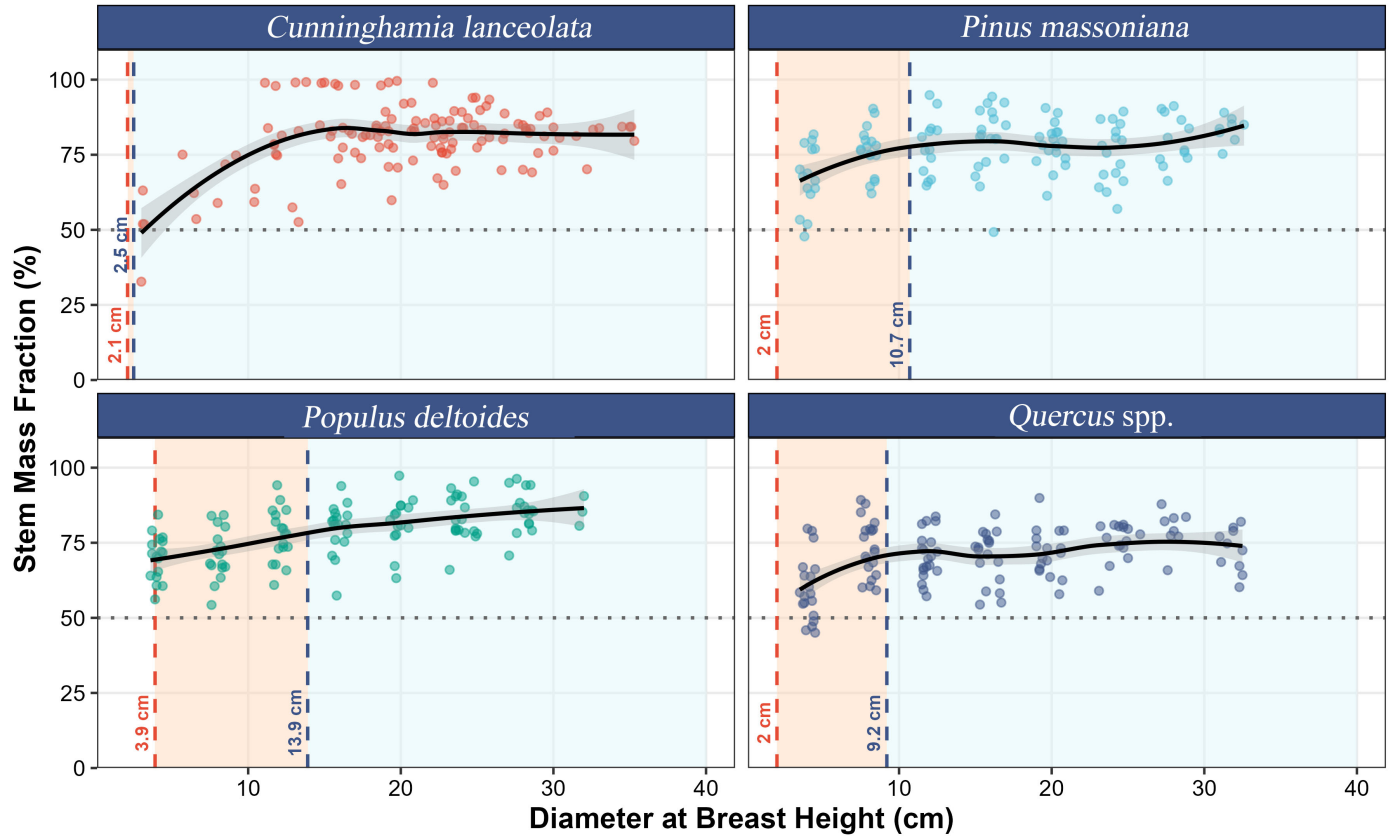
Ontogenetic trajectories of stem mass fraction (SMF) and critical management thresholds for the four taxa. Points are individual trees; solid lines are LOESS fits with 95% confidence intervals. The red dashed line marks the Crossover Point (*D*_CP_), where stem biomass exceeds crown biomass (SMF > 50%); the blue dashed line marks the Stabilization Threshold (*D*_ST_), where the marginal increase in SMF drops below 1%. The interval between them (orange zone) is the structural adjustment phase, the species-specific window for silvicultural intervention; the region beyond *D*_ST_ (blue zone) is the structural steady phase. Threshold values are annotated in each panel.

The non-linear trajectory of Stem Mass Fraction (SMF) quantified the dynamic transition from vegetative expansion to timber accumulation (**Figure 5**). The Crossover Point (*D_CP_*, red line) marks the critical threshold where stem biomass first exceeds the total crown biomass. For *C. lanceolata* and *P. massoniana*, this transition occurred at an extremely juvenile stage (*D_CP_* ≈ 2.0 cm), indicating that coniferous species establish absolute stem dominance during the seedling phase, thereby maximally compressing the preparatory period for volume growth.

The Stabilization Threshold (*D_ST_*, blue line) defines the onset of structural steady state. *P. deltoides* exhibited the broadest “Structural Adjustment Phase” (orange zone), with its *D_ST_* (13.9 cm) significantly lagging behind its *D_CP_* (3.9 cm). This 10-cm diameter interval corresponds to a steep ascent in SMF and high architectural plasticity, defining a critical window for silvicultural intervention: *P. deltoides* requires intensive water and nutrient management between DBH 4–14 cm to support its rapid structural remodeling and volume accumulation.

In contrast, the *D_ST_* (2.5 cm) for *C. lanceolata* nearly coincided with its *D_CP_* (2.1 cm). This extremely compressed or virtually absent adjustment phase reflects a rigid growth strategy. Consequently, the management focus for *C. lanceolata* must be shifted to early density control before canopy closure; once the seedling stage is passed, its fixed allometric pattern becomes difficult to regulate effectively through later silvicultural treatments.

## 4 Discussion

### 4.1 Biophysical mechanisms of variable selection and model compatibility

The screening of variables for biomass models serves fundamentally as a biophysical validation of the mechanisms controlling plant organ growth. Our study confirms that incorporating tree height (*H*) significantly reduces estimation errors for stem wood, supporting the geometric similarity hypothesis that stem biomass is strictly governed by the law of cylindrical volume (∝ *D*^2^*H*) (Dong et al., 2018; Zhang et al., 2016; Leverett et al., 2020). Conversely, crown components (branches and leaves) are decoupled from tree height, with DBH (*D*) consistently serving as the optimal predictor. This validates the applicability of Shinozaki’s pipe model theory to subtropical tree species—specifically, that the biomass of terminal photosynthetic organs is constrained by the sapwood transport area rather than stem height (Shinozaki et al., 1964). Furthermore, to address the logical paradox where “the sum of the parts does not equal the whole”—a common issue in independent fitting—we constructed a compatible equation system using NSUR technology. This approach physically captures the covariant relationships of biomass allocation among organs, providing a robust mathematical benchmark for refined multi-species biomass quantification (Parresol, 2001).

The taxon-dependent variation in optimal model form is itself ecologically informative rather than a mere technical artifact. Bark biomass of the broadleaved species was best predicted by the volume term D²H, implying that bark construction is coupled to stem volume growth, whereas conifer bark was better described by DBH alone, consistent with bark accretion driven mainly by radial (cambial) expansion and largely decoupled from height growth. The consistent selection of DBH for branches and leaves across all taxa reinforces the pipe-model expectation that terminal-organ mass is governed by sapwood cross-sectional area. These cross-taxon and cross-component differences in model structure therefore encode genuine differences in how species integrate diameter and height information into their allocation strategies. Although adopting component-specific predictors rather than a single system-wide form introduces minor heterogeneity in equation structure, additivity is enforced by the NSUR system regardless of the individual component forms; this component-level optimization thus improves biological interpretability and fit at no cost to the compatibility of the overall system.

Interpreted against Metabolic Scaling Theory (MST), our component-level results offer only partial support. Stem-wood biomass scaled almost isometrically with the volume term D²H (b ≈ 0.94–0.99), as expected from geometric volume constraints, whereas the leaf-versus-stem SMA exponents diverged markedly from the canonical 3/4 value: *P. massoniana* (b ≈ 0.79) and *Quercus* spp. (b ≈ 0.81) bracketed the MST prediction, *P. deltoides* fell below it (b ≈ 0.65), and *C. lanceolata* exceeded it (b ≈ 1.10). This indicates that a single universal exponent cannot capture component-level allocation across functional types, and that ontogenetic and life-history effects modulate departures from MST baselines.

### 4.2 Evolutionary trade-offs: vertical acquisition vs. structural persistence

The dynamic trajectories of biomass allocation during ontogeny quantify the evolutionary trade-off between “vertical space occupation” and “structural persistence” among different functional types. *P. deltoides* exemplifies the opportunistic strategy of pioneer species, characterized by a high stem allometric exponent (*b* ≈ 0.99) and an extremely early allocation turning point. By suppressing lateral branching and concentrating photosynthates into the main stem, *P. deltoides* achieves rapid vertical breakthrough at minimal structural cost, thereby establishing dominance in light competition (Poorter et al., 2012). This opportunistic allocation also helps explain the comparatively weak fit of the *P. deltoides* foliage equation (*R²* = 0.297): unlike stem and bark mass, foliage biomass is only loosely constrained by dendrometric variables and responds strongly to non-structural factors such as microsite fertility, stand density and competition, and phenological state, so that diameter or D²H alone cannot fully capture its variation.

In sharp contrast, *Quercus* spp. adopts a typical conservative strategy. It maintains high branch allocation throughout its life cycle, with a significantly delayed structural stabilization threshold. Although these high structural costs limit early stem accumulation rates, they enable the construction of high-density xylem and complex crown architectures, thereby maximizing mechanical safety margins and hydraulic transport efficiency. Consistent with the optimal partitioning theory proposed by McCarthy and Enquist (2007), this “structural persistence” strategy represents an evolutionary optimum for hardwood species to adapt to complex late-successional habitats and withstand environmental stress.

### 4.3 Biological validity of the generic *Quercus* complex model

**Figure 6:**
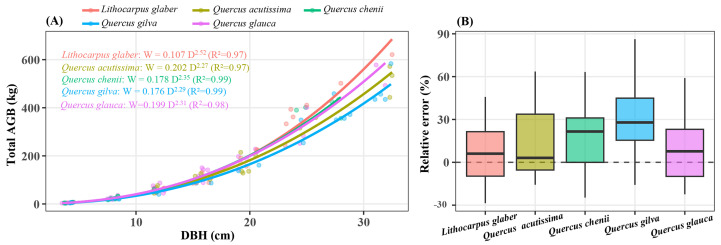
Evaluation of the generic model validity for *Quercus* spp. **(A)** Allometric trajectories of total aboveground biomass versus diameter at breast height (DBH) for five constituent species (*Lithocarpus glaber*, *Quercus acutissima*, *Q. chenii*, *Q. gilva*, and *Q. glauca*). Solid lines represent species-specific power-law fits, illustrating a high degree of morphological convergence among taxa. **(B)** Distribution of relative prediction errors generated by applying the generic *Quercus* spp. model to individual species. The dashed horizontal line indicates zero bias. Boxplots display the median (bold line), interquartile range (box), and variability (whiskers). Note that while the generic model provides unbiased estimates for core species like *Q. acutissima*, it systematically overestimates *Q. gilva* (positive relative error), indicating that this species accumulates less aboveground biomass at a given DBH than the genus-level average.

In regional forest inventories, developing a generic model for the highly species-rich *Quercus* spp. genus represents a critical challenge in balancing measurement precision with practicality. Our analysis of allometric trajectories (**Figure 6**) reveals that despite taxonomic differences among species such as *Q. acutissima*, *Q. glauca*, and *Q. chenii*, they exhibit a high degree of morphological convergence in the “biomass-DBH” dimension. This supports the biological rationality of grouping them into a single functional unit for modeling. Although residual analysis indicates minor systematic biases for high-density species (e.g., *Q. gilva*), the overall prediction error remains within a controllable range. This result suggests that for large-scale carbon accounting where species-level identification data is lacking, a generic equation based on the genus level can effectively eliminate uncertainties caused by species misidentification, offering a robust compromise solution (Chave et al., 2014; Paul et al., 2013).

Nevertheless, the systematic overestimation of *Q. gilva* by the generic model (**Figure 6B**, positive relative error) is directional rather than random, and therefore warrants explicit treatment in carbon accounting: at a given DBH, *Q. gilva* accumulates less aboveground biomass than the genus-level average, so applying the generic equation to stands rich in this species inflates estimated biomass and carbon stocks. This bias could be mitigated by introducing a species-specific downward correction factor for *Q. gilva*, thereby retaining the practicality of a single genus-level equation while curbing its largest source of error.

### 4.4 Differentiation in bark defense investment and implications for carbon accounting

The divergence in bark allometry between coniferous and broadleaved species unveils overlooked defense investment strategies, holding significant implications for precise forest carbon accounting. We found that, unlike the significant diminishing marginal investment observed in conifers (*b* < 0.9), *Quercus* spp. exhibits a rare isometric growth pattern (*b* ≈ 1.0), implying that bark accretion remains synchronized with timber accumulation. This finding challenges the simplified assumption in traditional carbon accounting that views bark as a fixed proportion. It confirms the defensive strategy of *Quercus* spp., which constructs a “thick-bark defense system” through continuous carbon investment to cope with fire disturbances or pathogen invasion. In mature forest carbon stock assessments, neglecting this allometric specificity will lead to a systematic underestimation of the bark carbon pool in broadleaved forests. Concretely, we recommend that operational forest inventories and biomass-expansion-factor (BEF) systems (i) record bark thickness or include a separately fitted bark component for hardwood taxa, and (ii) replace the assumption of a constant bark fraction with taxon-specific bark equations or correction factors — in particular an isometric (b ≈ 1.0) bark model for *Quercus* spp.— so that the bark carbon pool of mature broadleaved stands is not systematically undercounted. Therefore, future carbon accounting systems must transcend the single-volume perspective and develop refined measurement modules that incorporate differences in bark defense investment, thereby enhancing the accuracy of evaluating forest ecosystem carbon sequestration capacities.

## 5 Conclusion

By integrating biophysically constrained variable selection with a compatible equation system, this study resolves the long-standing issue of statistical non-additivity in biomass modeling, establishing a rigorous mathematical benchmark for precise regional carbon accounting. Our contribution is three-tiered — methodological (an additive NSUR equation system that resolves the sum-of-parts paradox), mechanistic (the quantification of divergent allometric life-history strategies), and applied (the implications of component-specific, especially bark, allometry for carbon accounting and stand management). More fundamentally, our analysis of allometric trajectories quantifies the evolutionary trade-offs in resource allocation among key tree taxa. *P. deltoides* and coniferous species pursue a “vertical maximization” strategy, securing rapid spatial dominance through early stem establishment and reduced investment in non-photosynthetic tissues. Conversely, *Quercus* spp. adheres to a “structural persistence” strategy, prioritizing substantial carbon allocation to branches and bark to construct a highly defensive canopy architecture. The discovery of isometric bark scaling in *Quercus* spp. challenges the conventional assumption of constant bark ratios; overlooking this component-specific allometry inevitably leads to a systematic underestimation of carbon stocks in mature broadleaved forests. Consequently, future forest management and carbon accounting must transcend the simplistic volumetric perspective, shifting towards a refined framework that integrates full-component allometric specificities to accurately evaluate the carbon sequestration potential and ecological stability of forest ecosystems.

## Data Availability

Publicly available datasets were analyzed in this study. This data can be found here: https://doi.org/10.5281/zenodo.18265796.
