# Supplementary material for: Divergent allometric strategies and biomass allocation patterns of four key tree taxa in subtropical China
Source: Front Plant Sci. 2026 Jun 29;17:1855123. doi: 10.3389/fpls.2026.1855123 (PMC13357904; doi:10.3389/fpls.2026.1855123)
Supplement: Supplementary file 1 [file DataSheet1.docx]

Figure S1. Residual diagnostics for the heteroscedasticity correction. (A) Raw residuals versus fitted values from ordinary least squares (OLS); the funnel-shaped spread (red LOESS trend) indicates variance increasing with size, violating homoscedasticity. (B) Standardized residuals after weighted least squares using *w* = 1/*X^k^*, with optimal exponents *k* = 1.70, 2.34, 3.17, and 5.08 for stem wood, bark, branches, and leaves, respectively; the uniform horizontal spread confirms that heteroscedasticity was removed.


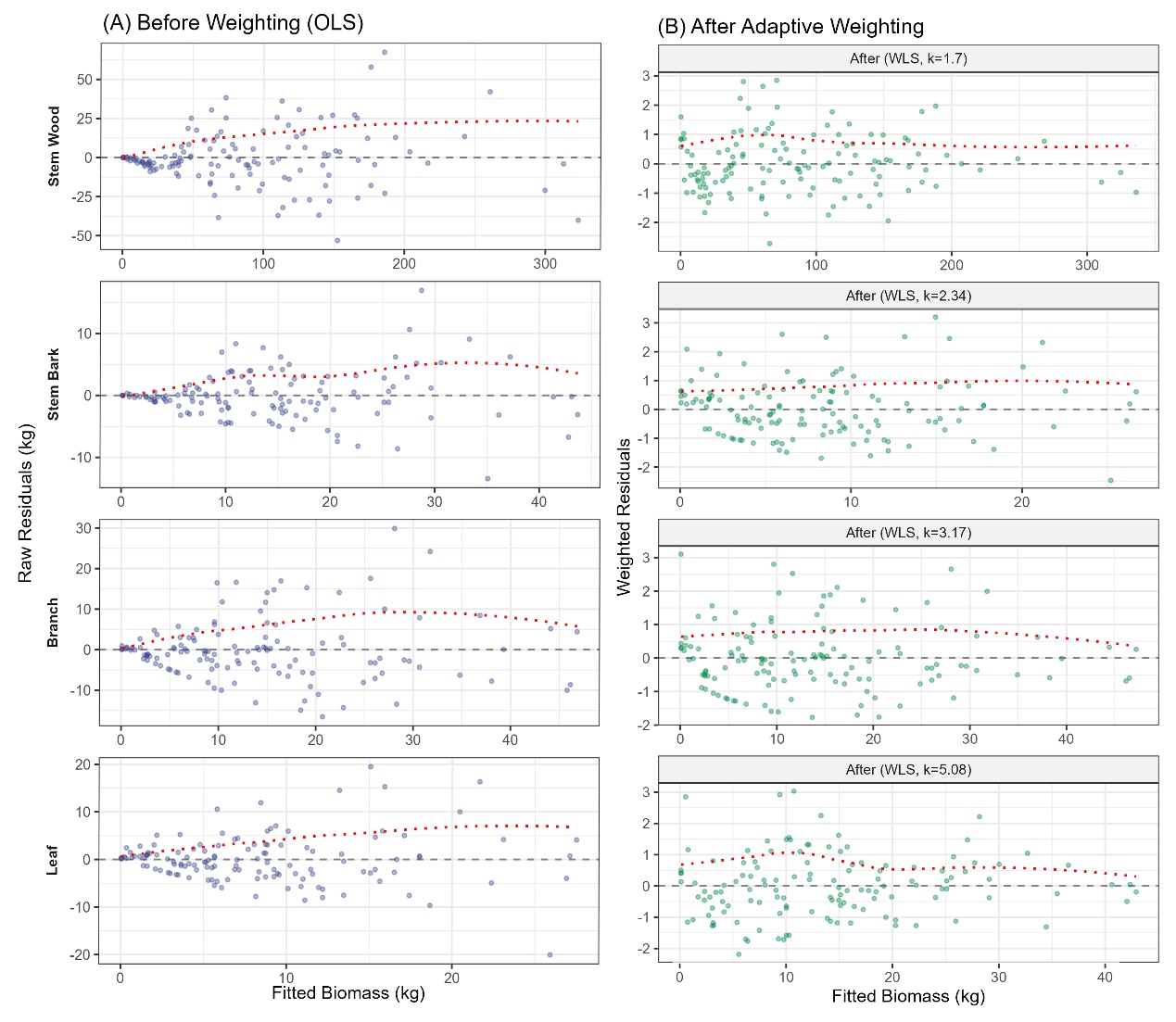


**Table S1.** Goodness-of-fit statistics for the three candidate base models across all taxon–component combinations. Model forms: M1, *W* = β₀*D*^β₁^; M2, *W* = β₀(*D*²*H*)^β₁^; M3, *W* = β₀*D*^β₁^*H*^β₂^. RSS, residual sum of squares; MAE, mean absolute error (kg); AIC, Akaike information criterion (lower is better); RMSE, root mean square error (kg). The model form adopted for each component was selected by jointly considering AIC, RMSE, and parameter plausibility (Section 2.3.1).

| Species | Component | Model | RSS | MAE | AIC | RMSE |
| --- | --- | --- | --- | --- | --- | --- |
| *Cunninghamia lanceolata* | Wood | M I | 47922.90 | 13.56 | 1143.20 | 19.20 |
|  | Wood | M II | 40988.03 | 12.20 | 1122.88 | 17.76 |
|  | Wood | M III | 37777.54 | 11.74 | 1114.28 | 17.05 |
|  | Bark | M I | 1919.30 | 2.64 | 724.91 | 3.84 |
|  | Bark | M II | 2211.62 | 2.84 | 743.34 | 4.12 |
|  | Bark | M III | 1893.95 | 2.64 | 725.18 | 3.82 |
|  | Branch | M I | 7213.80 | 5.16 | 897.03 | 7.45 |
|  | Branch | M II | 8741.43 | 5.56 | 922.00 | 8.20 |
|  | Branch | M III | 6847.15 | 5.11 | 892.25 | 7.26 |
|  | Leaf | M I | 3363.57 | 3.51 | 797.84 | 5.09 |
|  | Leaf | M II | 3755.74 | 3.63 | 812.18 | 5.37 |
|  | Leaf | M III | 3316.32 | 3.49 | 798.00 | 5.05 |
| *Pinus massoniana* | Wood | M I | 83574.05 | 17.10 | 1097.90 | 26.61 |
|  | Wood | M II | 46499.13 | 12.23 | 1034.40 | 19.85 |
|  | Wood | M III | 46037.44 | 12.24 | 1031.30 | 19.75 |
|  | Bark | M I | 838.78 | 1.59 | 572.30 | 2.67 |
|  | Bark | M II | 1190.84 | 1.91 | 613.65 | 3.18 |
|  | Bark | M III | 827.90 | 1.59 | 572.76 | 2.65 |
|  | Branch | M I | 9574.80 | 6.15 | 859.62 | 9.01 |
|  | Branch | M II | 15022.50 | 7.92 | 912.77 | 11.28 |
|  | Branch | M III | 6188.83 | 5.01 | 810.13 | 7.24 |
|  | Leaf | M I | 1451.11 | 2.00 | 636.98 | 3.51 |
|  | Leaf | M II | 1660.71 | 2.26 | 652.90 | 3.75 |
|  | Leaf | M III | 997.76 | 1.63 | 594.78 | 2.91 |
| *Populus deltoides* | Wood | M I | 41052.35 | 11.73 | 1069.74 | 18.27 |
|  | Wood | M II | 12990.72 | 6.31 | 928.22 | 10.28 |
|  | Wood | M III | 12119.23 | 6.10 | 921.67 | 9.93 |
|  | Bark | M I | 585.89 | 1.40 | 547.06 | 2.18 |
|  | Bark | M II | 320.80 | 1.02 | 472.97 | 1.61 |
|  | Bark | M III | 315.24 | 1.00 | 472.82 | 1.60 |
|  | Branch | M I | 7160.56 | 4.76 | 854.95 | 7.63 |
|  | Branch | M II | 8192.47 | 5.09 | 871.51 | 8.16 |
|  | Branch | M III | 6886.64 | 4.79 | 852.15 | 7.48 |
|  | Leaf | M I | 459.67 | 1.31 | 517.21 | 1.93 |
|  | Leaf | M II | 492.24 | 1.39 | 525.63 | 2.00 |
|  | Leaf | M III | 343.56 | 1.15 | 483.40 | 1.67 |
| *Quercus* spp. | Wood | M I | 108306.87 | 19.00 | 1223.47 | 29.20 |
|  | Wood | M II | 34465.52 | 10.52 | 1078.06 | 16.47 |
|  | Wood | M III | 33774.49 | 10.40 | 1077.49 | 16.31 |
|  | Bark | M I | 8963.89 | 4.82 | 907.02 | 8.40 |
|  | Bark | M II | 5584.66 | 3.83 | 846.93 | 6.63 |
|  | Bark | M III | 5148.87 | 3.87 | 838.61 | 6.37 |
|  | Branch | M I | 27667.77 | 8.95 | 1050.16 | 14.76 |
|  | Branch | M II | 35790.40 | 10.06 | 1082.85 | 16.79 |
|  | Branch | M III | 27301.39 | 8.92 | 1050.46 | 14.66 |
|  | Leaf | M I | 2493.79 | 2.93 | 744.54 | 4.43 |
|  | Leaf | M II | 3112.75 | 3.20 | 772.69 | 4.95 |
|  | Leaf | M III | 2140.77 | 2.67 | 727.15 | 4.11 |
